# In Defence of Machine Learning: Debunking the Myths of Artificial Intelligence

**DOI:** 10.5964/ejop.v14i4.1823

**Published:** 2018-11-30

**Authors:** Constance de Saint Laurent

**Affiliations:** a Department of Information Engineering, University of Bologna, Bologna, Italy

**Keywords:** artificial intelligence, machine learning, neural networks, learning, creativity, bias, ethics

## Abstract

There has been much hype, over the past few years, about the recent progress of artificial intelligence (AI), especially through machine learning. If one is to believe many of the headlines that have proliferated in the media, as well as in an increasing number of scientific publications, it would seem that AI is now capable of creating and learning in ways that are starting to resemble what humans can do. And so that we should start to hope – or fear – that the creation of fully cognisant machine might be something we will witness in our life time. However, much of these beliefs are based on deep misconceptions about what AI can do, and how. In this paper, I start with a brief introduction to the principles of AI, machine learning, and neural networks, primarily intended for psychologists and social scientists, who often have much to contribute to the debates surrounding AI but lack a clear understanding of what it can currently do and how it works. I then debunk four common myths associated with AI: 1) it can create, 2) it can learn, 3) it is neutral and objective, and 4) it can solve ethically and/or culturally sensitive problems. In a third and last section, I argue that these misconceptions represent four main dangers: 1) avoiding debate, 2) naturalising our biases, 3) deresponsibilising creators and users, and 4) missing out some of the potential uses of machine learning. I finally conclude on the potential benefits of using machine learning in research, and thus on the need to defend machine learning without romanticising what it can actually do.

Artificial intelligence (AI) and its latest advances have regularly made headlines over the past few years, giving the impression to many observers that the creation of a fully cognisant machine might be around the corner. The promises of AI, of building a perfectly objective, rational, and unfatigable intelligence, have indeed led to a variety of futuristic headlines, from those claiming that medical doctors would soon be obsolete, to the announcement that the first humanoid robot had been granted citizenship in Saudi Arabia. Beyond the more or less sensationalist reporting, claims that AI could solve many of humanity’s problems – from misinformation on social media to the administration of justice – have increasingly been gaining traction with the general public, following impressive advances made in computer science over the past decade. As more and more every day technological applications incorporate AI elements, however, concerns over its risks have also been mounting, most notably on issues of privacy – AI applications often requiring vast amounts of personal data – and of the control machines could one day exert over us – leading, for instance, Stephen Hawkins to warn us, in 2017, that AI could be the worst thing to ever happen to civilisation.

From the perspective of psychology and the social sciences, AI and its recent advances have been at the heart of two important debates. First, it has reignited discussions on whether machines can reproduce human cognition and what cognitive science could learn from them, in particular through the current popularity of neural networks. Frequent claims that neural networks have produced unexpected results – as if the machine had created or learned something new – have led some to believe that the possibility of a full-fledged cognisant agent emerging from the complex combination of ‘simple’ artificial neurons might not be that far-fetched after all. Second, the incorporation of smart machines in more and more areas of our lives has been at the origin of much research and debate on human-computer interactions, how we represent machines, and what may be the psychological and social consequences of the increasing presence of technology in our lives – from how care robots may be perceived to the role of AI in online misinformation.

The aim of this paper is thus to discuss and debunk some of the claims that have been made around the recent advances in AI, in particular when they relate to issues of concern to psychologists and social scientists. Ultimately, however, my aim is not to undermine the efforts of computer and data scientists, quite the contrary. Machine learning, the area of research that has produced most of the recent breakthroughs in AI, has proven, over the past few years, to have tremendous potential – some of which has already been realised, for instance, in the advances made in computer vision. But misconceptions as to what AI is and what it can currently do represent a twofold problem. First, they make having a debate about the real risks and benefits of using machine learning in different domains impossible. They leave the proper evaluation of the social and psychological consequences of using these technologies in the hands of the few who do understand how they work, and yet often lack the expertise of social scientists to fully foresee their consequences. Second, they may lead us to miss some of the most useful applications of machine learning, either because these misconceptions hide some of its potential, or because we end up throwing the baby with the bathwater, assuming that machine learning is as bad as the dangers represented by AI in some of its current application. The goal of this paper, therefore, is to defend the potential of machine learning, in particular for psychologists and social scientists, by debunking some of the myths surrounding artificial intelligence.

In a first section, I briefly summarise what AI and machine learning are, and how they work. The explanations are aimed at a general audience, and footnotes are added, where needed, to keep the text as light as possible. Based on this presentation, I discuss four main misconceptions around what AI can currently do: 1) It can create, 2) it can learn, 3) it is neutral and objective, and 4) it can solve ethically and/or culturally sensitive problems. In the third and final section, I argue that these misconceptions lead to four main dangers: 1) Avoiding debate, 2) naturalising our biases, 3) deresponsibilising creators and users, and 4) missing out some of the potential uses of machine learning.

Much of the arguments presented here are the result of numerous discussions with colleagues at psychology conferences over the summer of 2018. They have thus greatly benefited from their input.

## Artificial Intelligence and Machine Learning: A Very Brief Introduction

### Artificial Intelligence

AI can be defined as “a cross-disciplinary approach to understanding, modeling, and replicating intelligence and cognitive processes by invoking various computational, mathematical, logical, mechanical, and even biological principles and devices” (Frankish & Ramsey, 2014, p. 1). In general terms, the aim of AI is thus to develop machines able to carry out tasks that would otherwise require human intelligence. Behind this simple definition, however, hide many “misunderstandings caused by associations evoked by the name 'AI' [that] cannot be clarified by […] a definition” (Born & Born-Lechleitner, 2018, pp. vii–viii), “primarily because of our varied conceptions of intelligence” (Martínez-Plumed et al., 2018, p. 5180). Discussing the conflicting understandings of intelligence that permeate AI research is beyond the scope of this paper, but one distinction is useful here, that between ‘weak’ and ‘strong’ AI.

While weak AI refers to the development of algorithms able to reproduce or supplement human intelligence in very specific areas – from chess to image recognition – strong AI designates the attempts at creating machines capable of equalling or surpassing human intelligence in general – the creation of a fully functioning artificial mind or agent. Weak AI is already a reality, and it has very much been the case since the creation of the first computers. Whether strong AI is a possibility or not is, however, still a topic of debate. Although it is, at this point, more a theoretical and philosophical question than a technical one – we are still light years away from the robots of Asimov or of the more recent Marvel movies – it has permeated much of the discussion about what AI is already capable of doing or not. Indeed, partisans of a strong AI have tended to overestimate the significance of very local advances in the field, while others have remained more sceptical.

A striking example of this is the way different researchers have described the importance of Deep Blue, the IBM computer that defeated Gary Kasparov at Chess in 1997. Arguing that AI will soon be the engine of scientific discovery, autonomously formulating and testing hypotheses based on the existing literature, Kitano claimed that Deep Blue demonstrated that “a computer can exhibit human-level intelligence in a specific domain” (Kitano, 2016, p. 40). In contrast, others have described the same event by saying that although “there is no doubt that the AI Deep Blue II won […], it is still probably one of the dumbest software alive” (Borana, 2016, p. 66), in particular because of remaining doubts about whether “it was ever a fair fight to begin with” (*ibid*.) given that it had to be reprogrammed between every game. More recently, researchers have pointed out that Sophia, the first humanoid robot to be given a citizenship, is actually a poorly performing ‘chatbot’ with a face, and amounts more to a publicity stunt that anything else. Yet, it has made headlines around the world, often giving the impression that a fully functioning AI was around the corner.

What these examples point out is that the way both researchers and lay people represent AI and what it can do is often more the product of what we want to see in it than its actual capabilities. They also show that AI is, at times, a primarily ideological question rather than a clear-cut scientific or technical field within which specific or undebatable advances are being made. That said, there is little doubt that the field has indeed made major advances in the past few years, fuelled by increasing computing and memory capacities. This is particularly the case in the area of machine learning (ML) and artificial neural networks, a subfield of ML. Indeed, if the topic of AI has been the object of renewed passion and fantasy, it is in large part because of the recent promises of these two fields.

### Machine Learning

Machine learning was born at the meeting point between statistics – where it is often called statistical learning – and computer science – where the required computational algorithms have been developed. Simply put, ML refers to any statistical method where the parameters of a given model are ‘learnt’ from a dataset through an iterative process, usually to predict an output (a given value or category)^i^. This is done by minimising an error or loss function that calculates the difference between the values predicted by the model (based on the independent variables) and the values actually observed in the dataset (the dependent variable). In other words, the algorithm ‘learns’ how to best reproduce an existing dataset, in order to be used for categorisation or prediction in the future.

Because the models used in ML tend to be extremely complex, the exact parameters that will minimise the error function cannot be calculated directly, but only estimated through a gradient descent – an iterative process that looks at derivates to calculate in which direction the parameters should be updated, at each cycle, to lower the error rate. This ‘training’ of the model requires a large amount of labelled data (where the outcome is known), and this is all the more the case for particularly complex models, where a multitude of parameters needs to be estimated^ii^. Indeed, most ML methods use complex combinations of originally rather simple models – such as decision trees^iii^ or generalised linear models.^iv^ This allows researchers to simulate datasets that elude classical statistical methods, or to improve existing results. Unfortunately, while they can be extremely efficient, they often run the risk of being unintelligible, making their interpretation difficult and the detection of mistakes or biases, in some cases, almost impossible (Weld & Bansal, in press).

One of the biggest challenges posed by ML, however, is the sheer number of parameters and hyperparameters that need to be tuned in by researchers to appropriately fit or select a model, and that cannot be learnt during the training of the model. The non-exhaustive list includes choosing a type of model^v^ and its hyperparameters^vi^, what measures of error will be employed for training and for testing the model^vii^, how the parameters are to be updated^viii^ and how the data is to be fitted at each cycle^ix^, when to stop training the model^x^, and, finally on what basis to compare different models^xi^. This is, as with any other statistical modelling, after having decided which variables should be used and how in the first place.

As a result, the selection of the model is done, in the end, on the basis of the experience and more or less subjective impression of coherence of the researcher, who decides what models should be explored, how they should be deployed, and how they should be evaluated. In other words, ML requires the intervention of experts who tell the machine, very precisely, what it is that it needs to learn, and how it should learn it. The role of the machine, then, is to carry out calculation that would be much too long and much too complicated for researchers to perform themselves.

### The Example of Neural Networks

Artificial neural networks (NN) are one type of machine learning models particularly popular in AI applications, for two main reasons. First, they are extremely good at picking up complex patterns in the data to classify information and have been especially successful in areas such a computer vision (i.e., recognising images) or automated translations. Second, they require large amounts of data with great numbers of features (variables), as is exactly the case with big data. Recognising an image, for instance, involves manipulating data where each pixel is a feature,^xii^ and requires training sets of millions of labelled images, due to the large number of parameters that needs to be learned.^xiii^ In the case of big data, researchers are also confronted with a multitude of variables whose individual significance is often unclear as is, for instance, the significance of a single pixel in recognising that an image represents a cat. NN thus presents the great advantage of allowing researchers to input all the available data without crafting themselves more meaningful or simple variables.^xiv^

**Figure 1:**
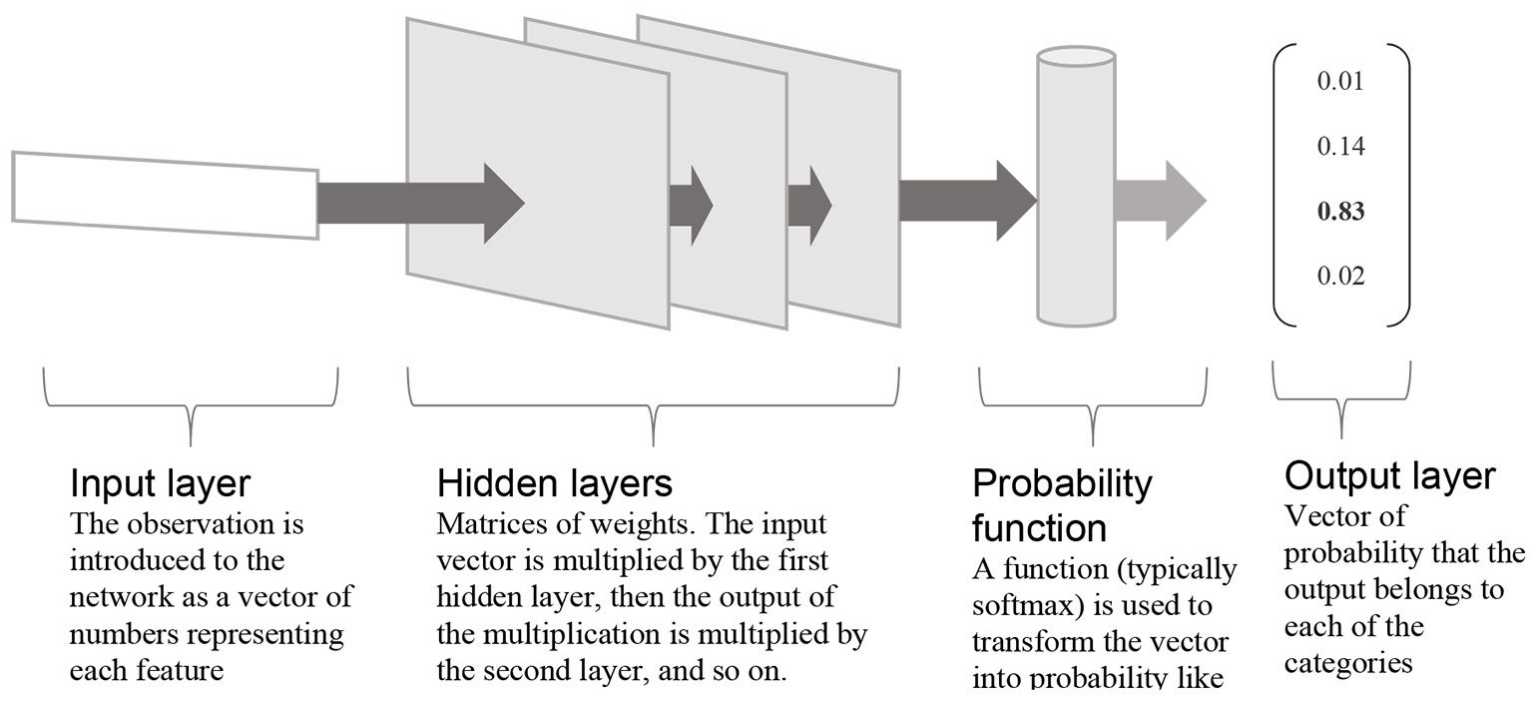
Example of an artificial neural network

NN are usually explained as working – as their name indicates – just like a simplified neural network. While this is one way of understanding them, and the analogy certainly inspired their creation, it is not the only one. Another way, illustrated in Figure 1, is to see them as a series of transformations of the data, each representing a ‘layer’ in the network. The transformations are most often linear: each variable is multiplied by a weight and the results are added together.^xv^ Each layer, then, is made of a series of weights by which the variables are multiplied. NN do also include some non-linear transformations, however, using more complicated functions. This is, at minimum, the case for the final layer in the case of a NN classifying observations (which is most often the aim of such a model). A function is then used, as a final layer, to transform the values calculated by the network into probability-like values, where 1) each possible category is given a probability between 0 and 1, and 2) all the probabilities add up to 1.

Globally, and without excessive oversimplification, an NN can thus be understood as the successive application of often relatively simple mathematical operations. As such, it has less in common with the biology of a human brain, and more with traditional statistical models. Their power, however, resides in the combination of these simpler operations in a multitude of large layers. And, in practice, NN lead to mathematical operations that are much beyond what could be calculated by hand – and, until quite recently, by a computer – for three main reasons.

First, as they use data with a large number of features, each of the layers of the network includes a proportionally large number of weights^xvi^. Second, they can be composed of many layers combined in complex ways – producing what are called deep neural networks. The NN used to translate text, for instance, are usually composed of two separate networks of dozens of layers, one for each of the languages, that are combined in the end to match the original text to its translation. Finally, the numerous weights on each of the layers of the NN need to be learn during a training phase, as with other machine learning models. This is done by using backpropagation, a method to update the parameters of nested models. The deeper and the more complex the model, however, the more difficult the outcome of backpropagation is to predict, and NN can produce unexpected results.^xvii^

For all of these reasons, neural networks represent a very good example of machine learning: it combines rather simple operations in often extremely complex models that are trained to learn to reproduce an existing dataset. As such, they also share the main advantages and disadvantages of machine learning methods. On the one hand, they can accurately model complex situations and automatically detect patterns that so far eluded humans and machines alike. On the other hand, they can produce quite unexpected results that are difficult to understand, even for the researchers who produced them. They also need a lot of fine tuning to find the best model and are in no way a magical method where the machine learns, on its own, to select the best possible model. Instead, they require the expertise and constant supervision of researchers.

While the general aim of this section on artificial intelligence, machine learning, and neural networks was to set the grounds for the arguments made in the rest of this paper, this lengthy and rather technical explanation has another objective. That is to highlight that these methods are, first and foremost, technical solutions developed by mathematicians, computer scientists, and statisticians in order to answer a simple question that, nonetheless, calls for a complex answer: how to best predict, given a series of information, the outcome of a situation or the category to which it belongs? This, in practice, allows us reproduce functions that are typically considered to be the product of human intelligence, for instance, inferences. However, claiming that these methods will one day lead to the elaboration of a fully functioning artificial mind is clearly jumping the gun. But could there be, already, signs that something ‘more’ is emerging from these complex combinations of simple operations? It is to this last question that I now turn.

## Common Misconceptions

In what follows, I review four common misconceptions surrounding what the algorithms and models used in artificial intelligence, machine learning, and neural networks can do: 1) they can create, 2) they can learn, 3) they are neutral and objective, and 4) they can solve ethically and/or culturally sensitive problems. Because they share some characteristics, they are considered in groups of two.

### AI Can Create and Can Learn

One of the claims on AI that has gathered the most interest recently is that it can create novelty, which has taken two main forms. First, several AI applications have made headlines, in the past couple of years, by showing that they could produce ‘original’ artwork in the style of known artists, with the first AI generated painting to be sold at an art auction last month gathering almost half a million dollars (Bryner, 2018). Second, some AI powered programmes have led to unexpected results, some of which may look quite creative. This was the case a few years ago, for instance, when Google released pictures of how its computer vision AI processed images, which looked like psychedelic artworks (Del Prado, 2015).

But can these examples be considered as forms of creativity – that is as producing something both novel and meaningful (Stein, 1953)? Some of the paintings produced by AI do seem like original works of known artists, but this is exactly where lies the rub: they have been trained to reproduce existing patterns, and these are simply recombined, randomly, in new forms. So far, however, no AI has (voluntarily) invented a new painting style. Similarly, the psychedelic pictures produced by Google’s image recognition AI are actually the results of the neural networks finding known patterns – like eyes or spirals – where there were sometimes none, resulting in odd but striking images. And, more importantly, if they appear to be meaningful – instead of the random outcomes we would expect of an unthinking machine – it is because researchers and journalists alike cherry-pick examples that do appear to be meaningful to them.

They could similarly focus on the many occasions where AI led to ridiculous or useless results, as when a model supposed to detect the malignancy of skin cancer found that pictures containing a ruler for scale were the most likely to be of cancerous lesions, or that the best way to build fast moving machines was to create giant devices and let them fall over (Day, 2018). AI can indeed lead to unexpected results, but this is because we do not always control perfectly the algorithms we create. And even in the best-case scenarios, AI does what it is programmed to do, which is not always what we want it to do. The creativity of algorithms, then, is very much in the eyes of those who produce and use them, and who select examples that are meaningful to them (for a discussion of the role of audiences in creativity, see Glăveanu, 2014).

There has also been, over the past few years, claims that algorithms can learn new things, and extrapolate results to new situations. After all, the most advanced AI models nowadays almost all use machine learning. However, the term is rather misleading: iteratively approximating the best parameters for a given statistical or mathematical model can hardly be considered as learning in the classical sense. Similarly, saying that AI learns from new situations to predict outcomes – to the point of being able to predict what you want before you even know it (Dormehl, 2018) – is simply restating, in anthropomorphic terms, what machine learning is set out to do. If an algorithm works well then, indeed, it will produce a model that can be used to predict the outcomes of novel situations; this is exactly why it was developed in the first place. This is, after all, one of the main aims of science: to develop, based on existing data, empirical and theoretical models that can be applied to new or more general situations. In some cases, it works, and the results can be extrapolated further. In others it does not, and new models have to be proposed. Focusing mainly on successful examples is here again cherry-picking.

It could be argued, however, that I am the one cherry-picking here: creativity does at times emerge out of randomness in humans too (e.g., Simonton, 1999), and learning can also fail to generalise to new situations. Is it possible that seeing AI as capable of creating and learning is not naively attributing anthropomorphic characteristics to a machine, but that, instead, refusing to recognise the abilities of AI is denying that humans are machines too – albeit biological ones? A thought experiment proposed by Searle in the 80’s is particularly useful in this regard. Indeed, Searle proposed that “a way to test any theory of the mind is to ask oneself what it would be like if one's own mind actually worked on the principles that the theory says all minds work on” (Searle, 2018, pp. 19–20).

Based on the descriptions made in the previous section of how machine learning and neural network work, how does it compare to the act of learning to recognise if a picture is of a cat or a dog – as you did as a child, for instance, when shown picture books – or of attempting to create a painting? Yes, the outcome of a neural network might be the same as what a human would produce, and it may sometimes be even more accurate or appropriate for the given task. But this does not mean in any way that the process is the same, with all of its consequences^xviii^: while for a human learning to recognise a cat or a dog means learning the concepts of cats and dogs, for a machine it simply means be able to recognise patterns of pixels and matching them to a certain category. As Searle (2018, p. 35) put it, the difference is:

Because the formal symbol manipulations by themselves don't have any intentionality: they are meaningless; they aren't even symbol manipulations, since the symbols don't symbolize anything. In the linguistic jargon they have only a syntax but no semantics. Such intentionality as computers appear to have is solely in the minds of those who program them and those who use them, those who send in the input and who interpret the output.

### AI Is Neutral and Objective, and Can Solve Ethically and/or Culturally Sensitive Problems

Another category of misconceptions about AI that has gathered much attention – and hope – is that it produces models that are neutral and objective, and thus can deal with complex social issues better than human beings would by removing our pesky biases. Indeed, some law scholars, such as Eugene Volokh, have argued that in the future justice could be carried out by AI, because it is possible to build models taking better decisions than human judges would, and that would yet display mercifulness and compassion (Waddell, 2018). In a similar vein, social media companies such as Facebook have invested sizeable resources in the development of AI automated content moderation, hoping to solve the major social and ethical challenges posed by this task and the search of a balance between freedom of speech and protection against abuse (Gillespie, 2018; Vaidhyanathan, 2018).

The main belief that underlies this position is that algorithms can produce results that are both neutral and objective. As naively put by Borana (2016, p. 65):

One of the major advantages of artificial intelligence is that its decisions are based on facts rather than emotions. Even after our utmost efforts, it is a well-known fact that human decisions are always affected in a negative way by our emotions.

While this is a rather extreme take on the neutrality of AI, it does reflect some of the underlying assumptions shared by both experts and lay people. As a result, some have argued that AI could actually be a solution to human bias – as assumed above by Borana – and could thus help us deal with situations that are known to have been riddled by prejudice. This has been the case, for instance, in candidate selection for jobs in the tech industry, where several companies have used AI models to help them choose future employees, with the belief that it would remove existing biases (Rosenbaum, 2018). In practice, however, it has resulted in exactly the opposite (Oppenheim, 2018).

This is because there are three fundamental issues with this view. First, it misunderstands the role of neutrality and objectivity in taking decisions, in particular when it concerns social issues. Indeed, it ignores the fact that knowledge always serves the interests of some over those of others, simply because it facilitates some actions more than others (Cornish & Gillespie, 2009). Choosing what information is relevant in a given context to take a decision is thus never neutral, and methods that simply attempt to maximise overall happiness have long shown their limits (e.g., Shaw, 1999). It also overlooks social and cultural differences, assuming that what is considered appropriate and desirable is the same everywhere. Yet, issues such as online content moderation, for instance, pose unique problems in different countries, where what is seen as obscene, violent, or dangerous can widely vary (Gillespie, 2018).

Second, AI models can only learn to reproduce existing classifications and they are, thus, at best, only as fair as humans have been in the past. In some cases, they may even lead to reinforced discrimination, by picking up on existing patterns and overly exploiting them to improve results. This is because the training data used can either be incorrectly labelled or tainted by historical biases (Hacker, in press). For instance, in the case of employment tools, they can rely on measures of performance where human coders have systematically underestimated the performance of people of colour or women, or on data where these categories are underrepresented, because they historically have been hired less by companies (ibid.). Models can quickly pick up on these patterns and reinforce them, as was for instance the case of Amazon’s AI recruitment tool, that learnt to badly evaluate resumes containing the word ‘woman’ (Oppenheim, 2018).

Third, AI models tend to lack transparency and intelligibility, making it difficult to measure exactly if they are discriminating and on what basis (Hacker, in press). Arguing for the need to create tools to make the results of AI more understandable, Weld and Bansal (in press) take the example of a study on the risk of developing pneumonia. By looking closely at the model constructed through machine learning, researchers discovered that it was predicting very low risks of pneumonia for asthmatic patients. This runs contrary to all other evidence and was due to the fact that many of these patients were already receiving treatment for lung diseases, which was not included in the model. This discovery was possible because the model used was still relatively simple, but the issue would have been much more difficult to spot with a neural network or a more complex machine learning model. This makes AI solutions almost impossible to apply in sensitive contexts: not only they may be picking on problematic variables, but they may have been developed on data that is too different from what it will encounter ‘in the wild’ (Weld & Bansal, in press). Because we do not always understand these models well, it is often extremely difficult if not impossible to predict whether they are indeed ecologically valid.

## The Dangers of Misunderstanding ML and AI

In this final section, and as a conclusion, I would like to highlight the four main dangers that the misconceptions outlined above represent: 1) avoiding debate, 2) naturalising our biases, 3) deresponsibilising creators and users, and 4) missing out some of the potential uses of machine learning.

The first danger brought by the myths surrounding AI is that it can lead us to believe that it will solve all or most societal issues we are currently confronted with, and thus that debate is unnecessary. Social media companies, for instance, and as mentioned above, have invested much hope and money in automated content moderation. Yet, AI cannot answer the questions raised by social media, such as what role these companies should play for societies; how they should be regulated and by whom; how they could strike a balance between freedom of speech and protection against abuse; or how and whether they should collaborate with states who are looking to police, for better or for worst, what their citizens say online. All these questions are, however, at the heart of content moderation, and our failure to collectively address them is at least partially responsible for the terrible impact social media has had on democracies and societies worldwide – from elections marred by misinformation in the US or Brazil, to the violent deaths hate-filled speech has led to in Myanmar or the Philippines (Gillespie, 2018; Vaidhyanathan, 2018). That is not to say that machine learning cannot help with content moderation, quite the contrary: once some guidelines and practices have been agreed on, models can be developed to scale the moderation to the millions of contents posted every day, and to avoid the trauma caused to content moderators by the worst the internet has to offer. But for this to be possible, a proper debate on the place social media has and should have in our societies needs to happen.

Second, believing that AI powered decisions are neutral and objective runs the risk of naturalising our biases, by making them appear as the product of a reasonable decision process. Arguments defending biased models already proliferate on internet forums: if AI discriminates against minorities and women, it is argued, it is only because the data proved that they are less efficient, trustworthy, or intelligent. The lack of social debate on these issues, as well as the tendency, in the Silicon Valley, to overlook social sciences and the critical perspectives they have produced (Cohen, 2017), are partially to blame for this kind of uncritical outlook. However, the blind belief that reason can be automated in AI and that it will produce an unbiased look at facts does have the lion share. Yet, as Hacker (in press, p. 35) has argued:

the application of machine learning to ever more important economic and societal decisions should not only be perceived as a risk, but also as a potential opportunity: of precisely constructing decision rules, of detecting and correcting discrimination with statistical and technical methods. […] Algorithmic fairness cannot, of course, address the roots of injustice in society, but it can mitigate some of its outcomes. This points to one great advantage of algorithmic vis-à-vis human decision making: its design parameters can be consciously chosen. The law of the algorithmic society only has to make the right choices.

Third, the misconceptions of AI highlighted in this paper have led to the deresponsibilisation of those who produce AI models as well as those who use them. By attributing too much agency to AI and giving it too much responsibility for learning and creating the models produced, we overlook the role of those who, after all, do select the models to build, decide how they should be built, for what purposes, which ones to keep, and whether they should be used or not. Yet, they can have tremendous consequences. When a team of data scientists at Stanford developed an algorithm capable of guessing, above chance, a person’s sexual preferences (Wang & Kosinski, 2017), they explained that their aim had only been to explore the potential dangers of AI (Levin, 2017). However, the choice to develop said algorithm – knowing that several countries around the world actively seek and prosecute people based on their sexual preferences – as well as the decision to present its results to media across the world, insisting on the high predictions rates in some categories – while overlooking the much more damning overall error rate – were all taken by the researchers, and to a certain extend by the journalists who reported on the story. Ultimately, the consequences of the use of AI are the responsibility of those who engineer it and of those who decide to deploy it. But overestimating the agency of AI models could deresponsibilise them, in particular in cases where AI is used to administrate justice, police populations, or evaluate risk. This is not to say that machine learning cannot play a positive role in these areas: it has been successfully used, for instance, by police investigators to comb through the large quantity of digital evidence they are now confronted with in criminal investigations (Mendick, 2018).

This brings us to the final danger that the misconceptions surrounding AI represent: that of overlooking the remarkable applications machine learning could have and, in some cases, already has. By overly considering AI in human terms – attributing it agency and reason – we forget that machine learning is first and foremost a brilliant tool to detect patterns in large amounts of data, enabling both prediction and recognition at levels unimaginable before. As such, it represents a unique tool not to replace human cognition, but to supplement it. Just as computers have revolutionised the way we work, machine learning will, if used properly, revolutionise the way we think. However, this cannot and should not be done by attempting to reproduce exactly human capabilities: it is precisely because word processing software operate completely differently than writing with a paper and pen does, or using a typewriter, that it is so useful – and also why most of us still use a paper and pen daily. Separating machine learning from the myths of AI, then, could ensure that we do exploit its potential to the fullest, and yet avoid falling into its pitfalls.

I do not believe that the future of machine learning is to replace researchers, asking the questions for us and answering them as well, conversely to what some have argued (Kitano, 2016). Algorithms will never know how to ask the right questions, to think critically about the literature, to formulate relevant and useful hypotheses, or to interpret results, in large parts because none of these tasks has a unique and universal answer. However, they can be used to analyse large and complex datasets, explore the exponentially growing literature most of us are confronted with, transcribe audio data, annotate videos, or thematically code text, to name only a few potential applications. That is, machine learning could be the unfatiguable assistant so many researchers wish they had, freeing us time to focus on the most interesting parts of research. Under the irreplaceable supervision of human experts, machine learning could thus revolutionise the way we do research, as so many other technological tools have in the past.
